# Familial Glucocorticoid Deficiency Type 4 Caused by a Novel Mutation in the Nicotinamide Nucleotide Transhydrogenase (NNT) Gene: A Clinical Report of Two Siblings

**DOI:** 10.7759/cureus.77046

**Published:** 2025-01-06

**Authors:** Ali S Alquraishi, Ahmed Albishri, Badriah G Alasmari, Walid Fawzy, Ali Hawan, Ahmed Al Zabali, Hamzah Al Qarni, Meshal Al Muqrin

**Affiliations:** 1 Pediatrics and Endocrinology, Armed Forces Hospital Southern Region, Khamis Mushait, SAU; 2 Pediatric Infectious Diseases, Armed Forces Hospital Southern Region, Khamis Mushait, SAU; 3 Pediatrics, Armed Forces Hospital Southern Region, Khamis Mushait, SAU; 4 Pediatrics, Armed Forces Hospital Alhada, Taif, SAU; 5 Pathology, Armed Forces Hospital Southern Region, Khamis Mushait, SAU; 6 Pathology and Laboratory Medicine, Armed Forces Hospital Southern Region, Khamis Mushait, SAU

**Keywords:** adrenal disorders, familial glucocorticoid deficiency, fgd, nnt mutation, primary adrenal insufficiency

## Abstract

Familial glucocorticoid deficiency (FGD) is a rare genetic disorder characterized by impaired cortisol production, resulting in primary adrenal insufficiency. Clinical manifestations include hyperpigmentation, hypoglycemia, failure to thrive, and recurrent infections, often triggered by stress. FGD is commonly inherited in an autosomal recessive manner, with various genetic mutations contributing to its pathogenesis. This case report discusses two siblings diagnosed with FGD type 4 caused by a novel mutation in a gene associated with adrenal steroidogenesis. The siblings presented with symptoms such as hyperpigmentation and hypoglycemic episodes and were treated with hydrocortisone, leading to significant clinical improvement. This case highlights the importance of genetic testing for early diagnosis, enabling effective treatment and prevention of severe complications. Long-term follow-up and education remain vital for managing this condition.

## Introduction

Familial glucocorticoid deficiency (FGD) is a rare inherited disorder characterized by the adrenal glands' inability to produce cortisol in response to adrenocorticotropic hormone (ACTH), leading to primary adrenal insufficiency [1]. Clinically, FGD presents with symptoms like hyperpigmentation, hypoglycemia, failure to thrive, and recurrent infections, which worsen during physical stress or illness [1]. Untreated, it can cause severe complications such as chronic hypoglycemia-induced neurological damage or fatal adrenal crises [2,3]. While hyperpigmentation is a hallmark feature due to ACTH overstimulation of melanocortin 1 receptors (MC1R), some patients may lack this sign due to mutations in related melanocortin pathways [2]. The disorder follows an autosomal recessive inheritance pattern and is caused by diverse genetic mutations [1,2].

The genetic basis of FGD is increasingly recognized as heterogeneous, involving mutations in multiple genes critical for adrenal steroidogenesis and related pathways [1,2]. Initially, mutations in the melanocortin 2 receptor (MC2R) and its accessory protein MRAP were predominant, but recent discoveries have broadened the spectrum of implicated genes [4]. For example, mutations in the nicotinamide nucleotide transhydrogenase (NNT) gene, essential for mitochondrial redox balance, have been linked to a novel form of FGD [5].

This case report discusses two siblings diagnosed with FGD Type 4 due to a previously unreported homozygous NNT mutation. Their cases underscore the importance of genetic testing in adrenal insufficiency and reveal the role of mitochondrial dysfunction in FGD pathophysiology.

## Case presentation

Our present cases involve two siblings, an 18-year-old male and his six-year-old sister, both diagnosed with FGD Type 4 caused by a novel mutation in the NNT gene. These cases highlight the autosomal recessive inheritance pattern of this rare disorder and underscore the importance of early diagnosis, genetic testing, and proper management to prevent adrenal crises.

Case 1

The first case involved an 18-year-old male patient, who was diagnosed at seven years of age with FGD Type 4 following the presentation of hyperpigmentation and hypoglycemic seizures. His past medical history reveals that skin darkening was first noticed at four months of age. At 3.5 years of age, he was presented to another hospital with hypoglycemic seizures, but adrenal insufficiency was not considered at that time. He initially presented to our hospital with symptoms of hypoglycemia, which were accompanied by darkened skin, especially on his hands, palms, oral mucosa, and genitalia, a finding that raised suspicion for adrenal insufficiency. Initial serum cortisol measured early in the morning was <0.5 nmol/L, and ACTH was initially 947 pg/mL. Serum electrolytes, including sodium and potassium, were normal. Renin, aldosterone, and 17-hydroxyprogesterone were within normal limits. Adrenal cortex antibodies were negative. These findings, along with his clinical presentation, confirmed the diagnosis of primary adrenal insufficiency. Genetic testing through whole exome sequencing (WES) revealed a homozygous mutation in the NNT gene, specifically a T-to-C substitution at c.1025, which results in a valine-to-alanine substitution at position 342 (p.Val342Ala).

Treatment was initiated with hydrocortisone (HC) at 14 mg/m²/day, and subsequent adjustments were made over the years to maintain control of ACTH levels. Initially, ACTH was measured at 947 pg/mL and dropped to 86 pg/mL after hydrocortisone was increased to 18 mg/m²/day. As he reached adolescence, his growth and development remained on track, and he did not experience any significant adrenal crises. His hydrocortisone dose was adjusted over time, based on clinical response and ACTH levels. To date, he has had no major complications related to the disease and continues to be followed up for adrenal function monitoring.

Case 2

The second case involves a six-year-old female, who was diagnosed with FGD type 4 at one year and seven months of age after presenting with skin hyperpigmentation on the palms, oral mucosa, and genitalia. Her condition was suspected to be due to a family history of FGD, as her older brother had been diagnosed with the same disorder several years earlier. Her clinical presentation included generalized hyperpigmentation that had been present for about four months, along with a history of recurrent infections. She was initially admitted to the hospital at the age of one due to gastroenteritis and hypoglycemia, responding quickly to intravenous fluids and feeding. Given her brother's known diagnosis, screening for adrenal insufficiency was performed, revealing low serum cortisol levels (28.4 nmol/L) and elevated ACTH (2050 pg/mL). The diagnosis of primary adrenal insufficiency was confirmed, and hydrocortisone (HC) therapy was initiated at a dose of 11 mg/m²/day.

Genetic testing confirmed the same homozygous mutation in the NNT gene found in her brother. Over the course of treatment, her hyperpigmentation resolved, and her ACTH levels gradually decreased, but remained elevated. The hydrocortisone dose was adjusted based on her clinical progress, and by the age of four years, her ACTH levels dropped significantly to 128 pg/mL. At six years of age, she continues to do well clinically. To date, her growth is within normal limits, with a height of 114 cm (> 50th percentile) and weight of 17 kg (25th percentile). Her serum electrolytes have remained stable, with normal levels of Na and K, and she has had no episodes of adrenal crisis. Her current dose of hydrocortisone is 13.5 mg/m²/day, with regular follow-up every three months to monitor her growth and adrenal function.

Family history and genetic analysis

Both siblings share a homozygous mutation in the NNT gene, identified through whole exome sequencing (WES) (Table 1). The mutation is a T-to-C substitution at c.1025, leading to a valine-to-alanine substitution at position 342 (p.Val342Ala). This mutation causes a defective nicotinamide nucleotide transhydrogenase enzyme, impairing cortisol production and resulting in ACTH resistance despite elevated ACTH levels. Both children inherited one defective allele from each parent, consistent with the autosomal recessive inheritance of the disorder. Early diagnosis in both cases was facilitated by genetic testing, which also guided treatment decisions.

**Table 1:** Clinical presentation and genetic diagnosis of both siblings FGD: familial glucocorticoid deficiency; ACTH: adrenocorticotropic hormone; NNT gene: nucleotide transhydrogenase gene

Parameter	Brother (18 years of age)	Sister (6 years of age)
Age at diagnosis	7 years	1 year, 7 months
Family history	Family history revealed similar cases in paternal cousins (one boy and one girl) with the same condition	Family history revealed a known case of FGD in her brother plus the cases in her paternal cousins
Presenting symptoms	Dark pigmentation, hypoglycemic seizures	Dark pigmentation on skin, mucous membranes, genitalia
Cortisol level (basal)	< 0.5 nmol/L	28.4 nmol/L
ACTH level	947 pg/mL	2050 pg/mL
Genetic diagnosis	Homozygous c.1025T>C in the NNT gene	Homozygous c.1025T>C in the NNT gene
Electrolyte profile	Normal serum electrolytes	Normal serum electrolytes
Initial treatment	Hydrocortisone 14 mg/m²/day, increased to 18 mg/m²/day over time	Hydrocortisone 11 mg/m²/day, adjusted to 13.5 mg/m²/day
Additional considerations	Follow-up for growth and adrenal function	Instructed on sick-day management and hydrocortisone use

Management and follow-up

Both siblings are regularly followed to monitor growth and prevent adrenal crises (Table 2). Treatment involves hydrocortisone replacement therapy, with dose adjustments based on clinical symptoms, ACTH levels, and growth patterns. Parents of both children were educated on the importance of sick-day protocols, including the need for stress-dose hydrocortisone during periods of illness or physical stress. Hydrocortisone doses are adjusted accordingly during these times to prevent potential adrenal crises, which can be life-threatening in patients with adrenal insufficiency. Both children have shown normal growth and development over time, and neither has experienced significant complications related to their condition, such as infections or adrenal crises. Ongoing follow-up continues with regular assessments of ACTH and serum electrolytes to ensure optimal management of their condition.

**Table 2:** Treatment, follow-up, and growth parameters ACTH: adrenocorticotropic hormone; HC: hydrocortisone

Follow-up period	Brother	Sister
Age	18 years (ongoing follow-up)	6 years (ongoing follow-up)
Height	175 cm (> 75th percentile)	114 cm (> 50th percentile)
Weight	68 kg (> 50th percentile)	17 kg (25th percentile)
Hydrocortisone dose	18 mg/m²/day	13.5 mg/m²/day
ACTH levels	ACTH levels were initially 947 pg/mL and dropped to 86 pg/mL after hydrocortisone was increased to 18 mg/m²/day.	ACTH dropped from 2050 pg/mL to 128 pg/mL
Cortisol levels	Normal cortisol levels (dependent on HC dosage)	Baseline cortisol normalized with treatment
Skin pigmentation	No signs of hyperpigmentation	No hyperpigmentation at last follow-up
General condition	Clinically stable, no crises or significant complications	Clinically stable, normal development
Sick-day management	Instructed on hydrocortisone use during illness or stress	Instructed on hydrocortisone use during illness or stress

## Discussion

FGD is a genetically heterogeneous disorder, with mutations identified in several key genes involved in adrenal hormone production. Traditionally, FGD has been associated with defects in MC2R or its co-chaperone MRAP, which are responsible for mediating ACTH signalling in the adrenal cortex [2,4]. Mutations in these genes lead to impaired cortisol synthesis despite elevated ACTH levels, a hallmark of the disease. In addition to MC2R and MRAP mutations, defects in steroidogenic acute regulatory proteins (StAR) in both siblings, which play a critical role in cholesterol transport into the mitochondria for steroidogenesis, have also been implicated in FGD [6]. However, these causes typically result in more severe forms of adrenal insufficiency and in some cases, may present with associated disorders such as lipoid congenital adrenal hyperplasia (LCAH) [7].

In recent years, the identification of additional genetic causes, including mutations in MCM4 and NNT, has broadened our understanding of FGD [8]. The novel mutation in the NNT gene identified in both siblings in this case report highlights the role of mitochondrial dysfunction in the pathogenesis of FGD [5]. NNT is critical for maintaining the redox balance within the mitochondria by catalyzing the reduction of NADP+ to NADPH [9]. This process is vital for protecting cells from oxidative stress [10]. In the adrenal cortex, where steroidogenesis is highly energy-dependent, NNT mutations lead to the accumulation of reactive oxygen species (ROS), which impair cortisol production by promoting mitochondrial damage and adrenal cell apoptosis [5]. This results in ACTH resistance, characterized by elevated ACTH levels without a corresponding increase in cortisol production, as seen in both siblings.

While mutations in NNT have been linked to FGD in a small subset of patients, the exact prevalence of NNT-related FGD remains unclear [5]. Our cases underscore the importance of genetic testing in diagnosing FGD, particularly when a family history of the condition is present. Both siblings in this report were diagnosed with the same homozygous mutation in the NNT gene (c.1025T>C, p.Val342Ala), confirming the autosomal recessive inheritance of the condition. The early diagnosis in the younger sibling was facilitated by the known diagnosis in the older sibling, highlighting the value of screening family members in cases of rare genetic disorders.

From a clinical perspective, the management of FGD involves lifelong glucocorticoid replacement therapy to correct cortisol deficiency. In both siblings, treatment with hydrocortisone was initiated early, and their doses were adjusted based on clinical response and laboratory monitoring of ACTH. Regular follow-up visits are essential to ensure optimal management, prevent adrenal crises, and monitor growth and development. In addition, education on "sick-day" management, including stress-dose glucocorticoid administration during illness or physical stress, is critical to preventing life-threatening complications in these patients [11]. Both siblings in this case report have responded well to treatment, with stable growth and no significant complications such as adrenal crises or infections. It is important to note that the suppression of plasma ACTH levels in FGD can be particularly challenging.

The identification of a homozygous mutation in the NNT gene in these two siblings also adds to the growing body of evidence suggesting that defects in mitochondrial function can contribute to adrenal insufficiency. This has important implications not only for understanding the pathophysiology of FGD but also for identifying potential therapeutic targets. Given that mitochondrial dysfunction is a central feature of this type of FGD, future research into antioxidants or other therapies aimed at mitigating oxidative stress may provide new avenues for treatment.

The cases of these two siblings also underscore the importance of genetic counselling for families affected by FGD. As this condition is inherited in an autosomal recessive manner, both parents are carriers of the mutated gene, and family members may also be at risk. Early identification through genetic testing can facilitate prompt treatment and prevent long-term complications, improving overall patient outcomes.

## Conclusions

FGD is a rare disorder with diverse genetic causes, including mutations in genes such as MC2R, MRAP, StAR, and more recently, NNT. The discovery of NNT mutations expands the genetic spectrum of FGD and highlights the role of mitochondrial dysfunction and oxidative stress in the development of adrenal insufficiency. Early diagnosis, particularly through genetic testing, is crucial for appropriate management and prevention of life-threatening complications such as adrenal crises. Regular follow-up and patient education are key to ensuring optimal care and long-term health for affected individuals.
